# Optimized Supercritical CO_2_ Extraction Enhances the Recovery of Valuable Lipophilic Antioxidants and Other Constituents from Dual-Purpose Hop (*Humulus lupulus* L.) Variety *Ella*

**DOI:** 10.3390/antiox10060918

**Published:** 2021-06-06

**Authors:** Nóra Emilia Nagybákay, Michail Syrpas, Vaiva Vilimaitė, Laura Tamkutė, Audrius Pukalskas, Petras Rimantas Venskutonis, Vaida Kitrytė

**Affiliations:** Department of Food Science and Technology, Kaunas University of Technology, Radvilėnų Rd. 19, 50254 Kaunas, Lithuania; nora.nagybakay@ktu.lt (N.E.N.); michail.syrpas@ktu.lt (M.S.); vaiva.vilimaite@ktu.lt (V.V.); laura.tamkute@ktu.lt (L.T.); audrius.pukalskas@ktu.lt (A.P.); rimas.venskutonis@ktu.lt (P.R.V.)

**Keywords:** hops, supercritical carbon dioxide extraction, hop bitter acids, flavor compounds, antioxidant activity

## Abstract

The article presents the optimization of supercritical CO_2_ extraction (SFE-CO_2_) parameters using response surface methodology (RSM) with central composite design (CCD) in order to produce single variety hop (cv. *Ella*) extracts with high yield and strong in vitro antioxidant properties. Optimized SFE-CO_2_ (37 MPa, 43 °C, 80 min) yielded 26.3 g/100 g pellets of lipophilic fraction. This extract was rich in biologically active α- and β-bitter acids (522.8 and 345.0 mg/g extract, respectively), and exerted 1481 mg TE/g extract in vitro oxygen radical absorbance capacity (ORAC). Up to ~3-fold higher extraction yield, antioxidant recovery (389.8 mg TE/g pellets) and exhaustive bitter acid extraction (228.4 mg/g pellets) were achieved under the significantly shorter time compared to the commercially used one-stage SFE-CO_2_ at 10–15 MPa and 40 °C. Total carotenoid and chlorophyll content was negligible, amounting to <0.04% of the total extract mass. Fruity, herbal, spicy and woody odor of extracts could be attributed to the major identified volatiles, namely β-pinene, β-myrcene, β-humulene, α-humulene, α-selinene and methyl-4-decenoate. Rich in valuable bioactive constituents and flavor compounds, cv. *Ella* hop SFE-CO_2_ extracts could find multipurpose applications in food, pharmaceutical, nutraceutical and cosmetics industries.

## 1. Introduction

Hops (*Humulus lupulus* L.), family Cannabaceae, are perennial herbaceous species of flowering plants valued in pharmacognosy for their sedative, antioxidant and antimicrobial properties [1]. These plants have been widely used as one of the essential ingredients in the brewing industry to confer organoleptic characteristics such as bitterness, aroma and taste to beers [2]. In line with market and consumer growing demands for natural products, currently, besides traditional sedative hop products (teas, extracts, tinctures, etc.), there is an increasing interest in innovative hop preparations based on their phytochemicals and bioactive potential [3]. Recent reviews highlight the diverse health-promoting effects of hops: anxiolytic, antioxidative, antidiabetic, anti-inflammatory, antitumor, anticarcinogenic and neuroprotective activities are attributed to the structural diversity of secondary metabolites present in hops [3,4,5,6]. As a result, hops and their extracts, besides their extensive use in the brewing industry that utilizes 90% of worldwide production, have started finding use in other food, nutraceutical, agricultural, pharmaceutical or cosmetic applications [3,4]. Therefore, there is an increasing interest in manufacturing high-quality hop-origin products, enriched with valuable essential oils, bitter acids and prenylflavonoids with the desirable organoleptic and bioactive properties [3,5,6,7]. 

As summarized in recent reviews, the recovery of valuable lipophilic *H. lupulus* fraction, primarily essential oils and hop bitter acids, can be achieved either conventionally by Soxhlet extraction or hydrodistillation, or assisted by intensifying technologies; for example, microwave-assisted hydrodistillation, pulsed electric fields, pressurized liquid extraction, etc. [4,7]. The ongoing demand for sustainable processes emphasizing environmental and safety aspects (i.e., avoiding harmful residual solvents in the extract) has incentivized the use of neoteric solvents and particularly supercritical fluids [8,9]. Among them, supercritical carbon dioxide extraction (SFE-CO_2_) of hops is a pioneer of commercial applications for this technique [7,9]. Supercritical CO_2_ hop extracts can serve both as valuable aroma bearing and high-bittering-potential bearing products, offering higher shelf life and bioactive compound stability than hop cones or pellets [3,7]. Generally, the commercial SFE-CO_2_ is performed at relatively low pressures (up to 15 MPa) as a one-stage process with liquid or supercritical CO_2_ to jointly recover aroma-rich fractions and a fraction of bitter acids from hops or their byproducts [7,10,11,12]. Moreover, aroma and bittering substances can be separated during two-stage SFE-CO_2_ to formulate different products to be used at the various stages of the brewing process [7]. However, prolonged extraction time is characteristic of the industrial SFE-CO_2_ of hops, while the products are basically tailored to the brewing industry’s needs [7,13,14]. 

Due to the constant development of novel *H. lupulus* extract applications [3], single-variety *H. lupulus* extracts with different properties and bioactive compound assemblies will be required to meet the demands of the functional food, nutraceutical, pharmaceutical and cosmetic industries. The so-called dual-purpose hop varieties, which are rich in essential oil and bitter acids [6], such as *Ella*, *Citrus*, *Columbus*, *Galaxy* and others, can serve as a promising feedstock for these purposes. Given our group’s interest in the utilization of emerging techniques to recover high added-value constituents from various feedstocks and agroindustrial residues [15,16,17,18,19], the present work is aimed at developing an effective SFE-CO_2_ process for production of natural dual-purpose hop extracts from *Ella* (previously named *Stella*) variety with high yield and strong oxygen radical scavenging capacity (ORAC). Towards this end, critical SFE-CO_2_ conditions, such as temperature, pressure and time were optimized via the response surface methodology (RSM) using central composite design (CCD) [20]. The phytochemical composition (flavor compounds, bitter acids and pigments) and in vitro antioxidant potential of the extracts, as well as target fraction recovery from hops, were further compared with the results obtained following the classical commercial one-stage SFE-CO_2_ approach. To the best of our knowledge, this is one of the first attempts to recover bitter acid and antioxidant-rich fractions from hops cv. *Ella* via SFE-CO_2_. Such bioactive-compound-rich single variety hop SFE-CO_2_ extracts could find multipurpose applications not only in brewing, but also in pharmaceutical, nutraceutical and cosmetics industries.

## 2. Materials and Methods

### 2.1. Hop Pellets

Dual-purpose hop cv. *Ella* T-90 pellets (further abbreviated as *Ella* hops), containing 7.1% moisture, 13.4% α-bitter acids and 1.40% essential oil, were obtained from the Baltic Brewing Supplies OÜ (Tallinn, Estonia). Before the extraction experiments, pellets were ground by an ultra-centrifugal mill ZM 200 (Retsch, Haan, Germany) using a 0.5 mm hole size sieve.

### 2.2. Chemicals

6-Hydroxy-2,5,7,8-tetramethylchroman-2-carboxylic acid (Trolox, 97%) and n-alkane standard solution C_7_-C_30_ (1000 μg/mL each component in hexane) were purchased from Sigma-Aldrich Chemie (Taufkirchen, Germany); 2-(3-hydroxy-6-oxo-xanthen-9-yl)benzoic acid, fluorescein (FL) and 2,2′-azobis-2-methyl-propanimidamide dihydrochloride (AAPH) were from Fluka Analytical (Bornem, Belgium); NaCl, KCl, KH_2_PO_4_ and K_2_S_2_O_8_ were from Lach-Ner (Brno, Czech Republic); Na_2_HPO_4_ was from Merck KGaA (Darmstadt, Germany); carbon dioxide and nitrogen gases (99.9%) were from Gaschema (Jonava, Lithuania). International Calibration Extract 4 (ICE-4), containing α- and β-acids (10.98% of cohumulone; 31.60% of humulone+adhumulone; 13.02% colupulone; 13.52% lupulone+adlupulone), was obtained from Labor Veritas AG (Zürich, Switzerland). Divinylbenzene/Carboxen/polydimethylsiloxane (DVB/CAR/PDMS) fibers and 20 mL SPME vials were purchased from Supelco (Bellefonte, PA, USA). All solvents were of analytical and HPLC-grade.

### 2.3. Supercritical CO_2_ Extraction (SFE-CO_2_) of Hop Pellets

*Ella* hop SFE-CO_2_ extracts were obtained under different experimental SFE-CO_2_ conditions in the SFT-110 extraction system (Supercritical Fluid Technologies, Newark, DE, USA) using 20.000 ± 0.002 g of ground hop pellets (0.5 mm), placed in a 50 mL cylindrical extractor (38 mm inner diameter, 136 mm length) between two layers of the cotton wool to avoid particle carryover to the system. To simulate currently used processes, the impact of the dynamic extraction (continuous flow of supercritical CO_2_) time (15–300 min; further abbreviated as τ) on the SFE-CO_2_ extract yield was determined at the fixed 10, 12.5 and 15 MPa pressure (further abbreviated as P) and 40 °C temperature (further abbreviated as T) combinations. In continuation, SFE-CO_2_ was further optimized at the higher pressure levels employing response surface methodology (RSM) using central composite design (CCD): P (25–45 MPa), T (40–60 °C); τ (30–90 min). Two response factors were selected for these optimization experiments: extract yield (RFI) and ORAC (RFII). The static extraction of 10 min was conducted prior to each dynamic extraction experiment based on the previously performed studies [15,16,17]. Constant extraction temperature was maintained by the surrounding heating cover of the extractor. The flow rate of CO_2_ was controlled manually by the micrometering valve and kept at 1.8–2.2 SL/min (standard liters per minute at standard state: P_CO2_ = 100 kPa, T_CO2_ = 20 °C, ρ_CO2_ = 0.0018 g/mL) during all experiments. The extracts were kept under the nitrogen flow for 10 min and stored in opaque bottles at −18 °C before the analysis. The yields of extracts were determined gravimetrically (±0.001 g) and expressed as g/100 g hop pellets (further abbreviated as HP). All extraction experiments were performed in duplicate.

### 2.4. Determination of In Vitro Oxygen Radical Absorbance Capacity (ORAC)

Following the procedure of Prior et al. [21] with some modifications, 25 µL SFE-CO_2_ extract solution in methanol (0.03 mg/mL MeOH) or MeOH (blank) was mixed with 150 µL of fluorescein solution (14 µmol/L) in the 96-well black opaque microplates, preincubated for 15 min at 37 °C, followed by rapid addition of 25 µL of AAPH solution (240 mmol/L) and fluorescence recording at every cycle (1 min × 1.1, a total of 120 cycles) using 485-P excitation and 520-P emission filters. Raw data were exported from the Mars software to Excel 2016 (Microsoft, Roselle, IL, USA), and the area under the fluorescence decay curve (AUC) was calculated as:

(1) $$AUC=1+\overset{i=150}{\underset{i=1}{\sum }}\frac{f_{i}}{f_{0}}$$

where *f*_0_ is the initial fluorescence reading at 0 min, *f*_i_ is the fluorescence reading at time *i*.

The results were expressed as mg Trolox equivalent antioxidant capacity per gram of SFE-CO_2_ extract or hop pellets (further abbreviated as TEAC_ORAC_, mg TE/g E or HP) using dose-response curves for Trolox (250–1500 µmol/L). Experiments were performed in quadruplicate.

### 2.5. Determination of α-and β-Acid Composition by UPLC-MSn Analysis 

For the quantitative and qualitative determination of hop bitter acids, 10 ± 0.001 mg of SFE-CO_2_ extracts was dissolved in MeOH and further diluted to a final concentration of 10 μg/mL and filtered through a polyamide filter into vials before UPLC-MSn analysis. The analysis was performed using an Acquity UPLC H-class system (Waters, Milford, MA, USA) combined with a Waters XEVO TQ-S mass spectrometer (Waters, Milford, MA, USA). The Acquity UPLC was equipped with a binary solvent delivery system, an autosampler with a column thermostat and a data station running the MassLynx 4.0 acquisition and data processing software. An Acquity BEH C18 column (1.7 µm, 100 × 2.1 mm, i.d.) was used to separate compounds. The mobile phase was initially composed of 50% eluent A (0.3% of formic acid in water) and 50% B (0.3% of formic acid in acetonitrile), followed by a linear increase of B from 50% to 100% in 7 min, then holding at 100% B for 1 min and finally equilibrating the column to initial conditions (50% of B) for 4 min. The eluent flow rate was 0.4 mL/min. The effluent was introduced directly into the mass spectrometer equipped with an ESI source. Compounds were monitored by their characteristic fragment ions: 349.16 → 225.08 for cohumulone and 363.16 → 239.02 for humulone in the positive ionization mode; 399.29 → 287.12 for colupulone and 413.29 → 301.13 for lupulone in the negative ionization mode. The capillary voltage was maintained at 3 kV, desolvation temperature at 350 °C, desolvation gas flow at 750 L/h, cone gas flow at 150 L/h, nebulizer pressure at 6 bar. Nitrogen was used as the desolvation and nebulizing gas. Argon was introduced into the collisional cell at a rate of 0.15 mL/min as the collision gas. The external calibration curve for α- and β-acid quantification was designed using ICE-4 standard at concentrations ranging from 2.5 to 30 μg/mL; results were expressed as mg/g E and HP. Experiments were performed in triplicate.

### 2.6. Determination of the Total Chlorophyll and Carotenoid Content

As previously described by Lichtenthaler and Buschmann [22], the total content of the selected supercritical CO_2_-soluble pigments (chlorophylls and carotenoids) was determined spectrophotometrically, measuring the absorbance of SFE-CO_2_ hop extracts (10 mg/mL acetone) at 662 nm, 645 nm and 470 nm wavelengths. The concentrations of chlorophyll A, chlorophyll B, total chlorophyll and total carotenoid content (μg/mL E) were calculated using the following equations of Mouahid et al. [23] and further expressed as μg/g E and HP (measurements performed in quadruplicate):

(2) $$C_{Chloropyll\;A}=11.24\times Abs_{662}-2.04\times Abs_{645}$$

(3) $$C_{Chloropyll\;B}=20.13\times Abs_{645}-4.19\times Abs_{662}$$

(4) $$C_{Carotenoids}=\frac{\left(1000\times Abs_{470}-1.90\times C_{Chlorophyll\;A}-63.14\times C_{Chlorophyll\;B}\right)}{214}$$

### 2.7. Determination of Volatile Compound Composition by SPME-GC×GC-TOF-MS Analysis

In order to determine the volatile compound composition, 0.100 ± 0.001 g of SFE-CO_2_ extracts was placed in a 20 mL SPME vial and subjected to the solid-phase microextraction (SPME) with a DVB/CAR/PDMS fiber at the following conditions: temperature 40 °C, equilibration time 15 min, extraction time 30 min. The analysis of SPME-derived samples was conducted on a comprehensive gas chromatography time-of-flight mass spectrometry (GC×GC-TOF-MS) LECO Pegasus 4D system, consisting of an Agilent 7890A GC system, a Gerstel multipurpose sampler MPS (Gerstel GmbH, Mulheim an der Ruhr, Germany) coupled with a high-speed TOF-MS detector (LECO, St. Joseph, MI, USA) and a four-jet cryogenic modulator (Zoex, Houston, TX, USA). The chromatographic system was made up of a primary column BPX-5 (30 m, 0.25 mm i.d., 0.25 μm film thickness) (SGE Analytical Science, Australia) linked with a secondary column, BPX-50 (2.0 m, 0.10 mm i.d., 0.1 μm film thickness). Working conditions were: desorption time 5 min; oven temperature started at 40 °C (hold 1 min) and ramped to 250 °C at 7 °C/min rate (hold 1 min); modulator offset temperature 15 °C; transfer line to MSD 250 °C; the GC injector port temperature set at 150 °C then ramped to 250 °C at 720 °C/min; carrier gas (He) 1 mL/min; split ratio 1:20; TOF-MS acquisition rate 10 spectra/s, mass range 30–550 m/z units; detector voltage 1550 V; ion source temperature 250 °C. Data from the GC×GC-TOFMS system were collected by ChromaTOF software v.4.22 (LECO) after a solvent peak delay of 360 s. Volatile compounds were identified by comparing their mass spectra with the Adams, NIST, MainLib and Replib mass spectral libraries (acceptable matches: signal-to-noise ratio >50 and similarity >750). The linear retention indexes (LRI) were calculated using the retention times of C_7_-C_30_ n-alkanes series and further compared with previously published data in literature [24,25,26,27,28,29,30], when available. The results were expressed as GC peak area arbitrary units × 10^7^ (further abbreviated as AU) and percentage (%) of the total GC peak area. Experiments were performed in triplicate.

### 2.8. Experimental Design

CCD-RSM was employed to identify optimal SFE-CO_2_ conditions by determining the effect of P (25–45 MPa), T (40–60 °C) and τ (30–90 min) on SFE-CO_2_ extract yield and ORAC, selected as the response factors (RF) in the optimization experiments. The face-centered CCD design with 8 factorial, 6 axial and 6 center points (in total, 20 experimental runs), randomized order of experiments, models and the second-order polynomial equations for both RFs were established using the Design-Expert 12 software (Stat–Ease Inc., Minneapolis, MN) as previously described elsewhere by our research group [15,16,17,18,19]. Student test (*p*-value) at 5% probability level (*p* < 0.05), “lack of fit” coefficient and the Fisher test value (F-value) were used to define the statistical significance and adequacy of the model and each variable for both RFs.

### 2.9. Statistical Analysis

Mean values and standard deviations were calculated using MS Excel 2016. GraphPad Prism 7.04 software (2017) was used to compare the means that showed significant variation (*p* < 0.05), applying one-way analysis of the variance (ANOVA), followed by Tukey’s post hoc test, and was used to calculate Pearson correlation coefficients (two-tailed, *p* < 0.05) between the selected phytochemical composition indices and TEAC_ORAC_ values.

## 3. Results and Discussion

### 3.1. Evaluation of SFE-CO_2_ of Hops at 10–15 MPa Pressure

**Figure 1:**
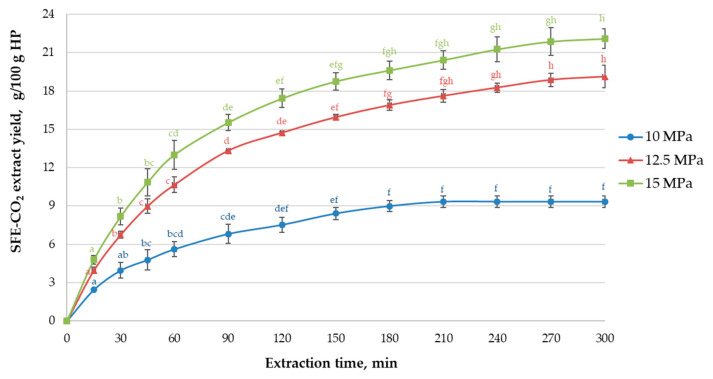
The influence of the extraction time on the *Ella* hop SFE-CO_2_ extract yield (g/100 g pellets) at 10–15 MPa pressure at 40 °C. SFE-CO_2_: supercritical carbon dioxide extraction. Different superscript letters indicate significant differences for each graph individually (one-way ANOVA and Tukey’s test *p* < 0.05).

SFE-CO_2_ is one of the most common non-conventional upscalable extraction techniques to isolate high-quality non-polar constituent assemblies from hops with relatively cheap, non-toxic, non-flammable, generally recognized as safe (GRAS) and readily eliminated after extraction food-grade solvent CO_2_ [7,31]. The effectiveness of SFE-CO_2_ in terms of cumulative yields and selectivity towards specific hop constituents can be achieved by modifying P, T and τ; other factors, such as particle size and cosolvent addition may also be important [32]. The task of this part of the study was to assess commonly used process parameters in hop extraction industry before further SFE-CO_2_ optimization experiments. The extraction curves in Figure 1 depict the cumulative *Ella* hop extract yield as a function of time in the one-stage process at 10–15 MPa and 40 °C, which is most commonly applied at the industrial level [7]. The yields and TEAC_ORAC_ values at the final point of the kinetic experiments (after 300 min) are reported in Table 1. 

All three extraction curves followed a similar pattern (Figure 1): ~50% of the final SFE-CO_2_ extract yield was obtained after 45 min of extraction, ~80% after 120 min. For 10 MPa, the equilibrium state was reached after 180 min, yielding 9.3 g/100 g of light yellow extract. For 12.5 and 15 MPa, ~96% of the final extract yields, amounting to 19.1 and 22.1 g/100 g (Table 1), respectively, were recovered after 240 min. Thus, the increase in CO_2_ density from 629 kg/m^3^ at 10 MPa to 780 kg/m3 at 15 MPa resulted in a remarkable (>2-fold) increase in yield. Nevertheless, the prolonged τ (>240 min) was required to achieve this aim, which is in agreement with the previous data for SFE-CO_2_ of hops at low P range (<20 MPa) [10,12,33]. It can be noted that the shape of *Ella* hop SFE-CO_2_ curves (Figure 1) is almost similar to the one previously reported for other hop varieties, exhibiting a rather long low-yield period at the beginning of extraction [10,33,34]. 

Antioxidant capacity of lipophilic extracts obtained at 10–15 MPa was assessed using biologically relevant peroxyl radical inhibition-based ORAC assay [35]. As reported in Table 1, the P change from 10 to 15 MPa augmented extract TEAC_ORAC_ by 21% (from 1252 to 1515 mg TE/g). Considering extraction yields, the calculated recovery of TE antioxidants from the pellets was in the range of 117–335 mg TE/g, indicating a nearly 3-fold increase due to the higher P. For applications in functional food, nutraceutical, pharmaceutical and cosmetic industries, the strong antioxidant potential of SFE-CO_2_ extracts is the desired quality characteristic. Thus, the extraction of hop antioxidants at higher yields could be considered a more efficient approach for such purposes, preferably within shorter times to reduce the operational costs of the process.

### 3.2. Evaluation of SFE-CO_2_ of Hops at 24–45 MPa Pressure

#### 3.2.1. Central Composite Design and Model Analysis

At the following steps, the research was targeted to increase SFE-CO_2_ yield and recover valuable constituents from *Ella* hops under significantly shorter extraction time. Since higher P (>25–30 MPa) accompanied by T of 50 ± 10 °C can substantially increase the yield of hop SFE-CO_2_ extracts [7], CCD-RSM was employed to optimize the SFE-CO_2_ process by testing different experimental conditions at the following P and T levels: P (25–45 MPa) and T (40–60 °C). The range of τ (30–90 min) was determined based on the several preliminary runs at the center P and T values (35 MPa, 50 °C), indicating negligible change in yield at τ > 90 min. Within the selected region of operability, total SFE-CO_2_ hop extract yield (RFI) ranged from 13.9 to 27.6 g/100 g HP, while the TEAC_ORAC_ (RFII) increased from 252 to 375 mg TE/g HP, both well-fitting the predicted values of the designed models (Table 2). Calculated Pearson correlation coefficient of 0.9363 (*p* < 0.0001) additionally indicated strong significant positive correlation between the SFE-CO_2_ yield and TEAC_ORAC_ under these conditions.

**Figure 2:**
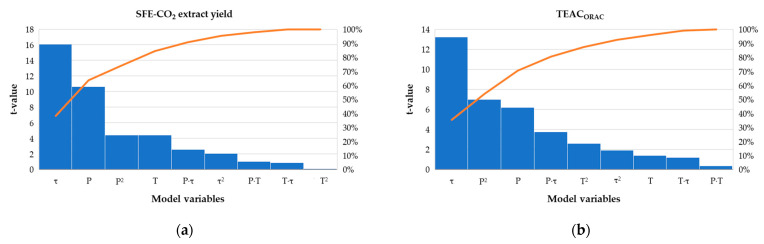
Pareto charts (*p* = 0.05) for the main effects of SFE-CO_2_ pressure (P), temperature (T) and time (τ) and interactions thereof on the *Ella* hop: (**a**) SFE-CO_2_ extract yield (g/100 g HP); (**b**) oxygen radical scavenging capacity (TEAC_ORAC_, mg TE/g HP).

Both models were reasonably reproducible with low variation coefficients (<3%), high determination coefficients (R^2^ > 0.96) and good agreement between the adjusted and predicted R^2^ values (difference < 0.20), additionally confirming the good fit of the model to the experimental data (Supplementary Materials, Table S1). Based on the ANOVA (Table 3), developed models were statistically significant (*p* < 0.05), with *F*-values of 51.71 and 35.20 for RFI and RFII, respectively. Time (τ) was the primary extraction parameter responsible for the observed changes in both extract yield (*F* = 258.72) and TEAC_ORAC_ (*F* = 175.15) under the different experimental conditions. The significance of other model terms for the extract yield decreased as follows: P < P^2^ (showing the non-linear concave relationship between P and RFI) < T < Pτ interaction. The influence of other interactions (PT and Pτ) and second-order terms (T^2^ and τ^2^) was not significant. Besides τ, TEAC_ORAC_ was mainly affected by the P^2^ and P, to a lower extent by Pτ and T^2^, while other factors and their interactions did not exert any significant input towards RFII. The Pareto charts (Figure 2) visualize these effects and indicate that three primary factors (τ, P and P^2^) together contributed to the >70% of the observed responses. 

The following second-order polynomial regression equations describe the empirical relationship between the independent model variables and selected response factors (in terms of coded factors):

(5) $$\begin{array}{ll} Yield_{SFE-CO_{2}} & =23.92+2.27\times P-0.94\times T+3.44\times \tau -0.25\times \left(PT\right)-0.61\times \left(P\tau \right)+0.20\times \left(T\tau \right)-1.80\times P^{2}\\ & \;+0.03\times T^{2}-0.82\times \tau^{2} \end{array}$$

(6) $$\begin{array}{ll} TEAC_{ORAC} & =344.65+15.61\times P-3.48\times T+33.49\times \tau +0.98\times \left(PT\right)-10.63\times \left(P\tau \right)-3.29\times \left(T\tau \right)-33.70\times P^{2}\\ & \;+12.50\times T^{2}-9.15\times \tau^{2} \end{array}$$

#### 3.2.2. Analysis of the Response Surface Plots 

Two-dimensional and three-dimensional response surface plots visualize the effects of the independent variables on the extract yield (Supplementary Materials, Figure S1) and TEAC_ORAC_ (Supplementary Materials, Figure S2). For example, the plots illustrating the effect of T and P at fixed τ of 60 min indicated that the amount of the extract and TEAC_ORAC_ did not exceed 20 g/100 g and 300 mg TE/g, respectively, at the minimal P (-1 level) of 25 MPa. Nevertheless, extraction at 25 MPa and 40 °C already after 30 min was more efficient than 180 min extraction at 10 MPa, amounting to 166 and 216% of the final 10 MPa yield and TEAC_ORAC_, respectively; the results for both responses after 90 min were higher by 5–43% than those measured for 12.5 and 15 MPa after 300 min (Table 1, Table 2). This may be explained by the substantially higher CO_2_ density and solvating power towards lipophilic constituents at 25 MPa and 40 °C (880 kg/m^3^) in comparison to 10, 12.5 and 15 MPa under the same extraction temperature (629, 705 and 780 kg/m^3^, respectively). 

The analysis of 2D and 3D response surface plots in Figure S1 (Supplementary Materials) also outlined that combinations of 37–42 MPa and 40–45 °C augmented the yields to the maximum values (>26 g/100 g) within the selected region of operability. Maximum yield values were also reached due to the prolonged τ (>75 min), as depicted in Figure S1a,b (Supplementary Materials). Although the CO_2_ diffusivity and solute vapor pressure are greater at higher temperatures [22], the yield reduction was observed >45 °C at all tested P levels. This can be explained by the decreasing solvent density due to the T increase from 40 to 60 °C: by 11% at 25 MPa (from 880 to 787 kg/m^3^), 8% at 35 MPa (from 935 to 863 kg/m^3^) and 6% at 45 MPa (from 975 to 913 kg/m^3^). Thus, the effect of density governs the retrograde behavior of T in the *Ella* hop extraction model. Moreover, even high-end experimental P (45 MPa) remains lower than the so-called crossover (inversion) P value, when the higher T would favor the extraction since increasing solute vapor pressure would outweigh the impact of decreasing CO_2_ density [32]. 

For TEAC_ORAC_, the plots with temperature and pressure effects at the fixed extraction time acquired a slight saddle shape, as presented in Figure S2a (Supplementary Materials). The highest values were reached at the 35–40 MPa and 40–43 °C combinations, which overlapped favorably with the optimal T and low-to-middle range of the desired P for the maximum yield. Based on TEAC_ORAC_, even higher amounts of radical scavengers were recovered by continuing extraction (τ > 80 min), especially at T < 45 °C, which is in agreement with the observations for the yield. 

#### 3.2.3. SFE-CO_2_ Optimization by the Desirability Function

Based on the response surface plots and the predictive equations that describe the model, the SFE-CO_2_ optimization was upgraded to obtain *Ella* hop extract combining a high yield (>26 g/100 g HP) and TEAC_ORAC_ (>360 mg TE/g HP) under the lowest possible P and shortest τ. For this task the Design-Expert software suggested 37–38 MPa, up to 43 °C, 80–85 min. For example, 80 min extraction at 37 MPa and 43 °C yielded 26.3 g/100 g HP of greenish-brown extract with the TEAC_ORAC_ of 1481 mg TE/g E, equivalent to 390 mg TE/g HP (Table 1). Good agreement between the experimental and the predicted values under deduced optimal conditions additionally confirmed the suggested model’s validity for both response factors (Supplementary Materials, Table S2). Generally, maximum extract yields from Ella hops at 10–37 MPa were higher than those previously reported under the various experimental conditions; e.g., for *Hallertau Mittelfrüh* pellets it was 7 g/100 g at 20 MPa/55 °C/180 min [10]; for *Nugget* variety and five Chilean hop ecotype pellets it was 3–13 g/100 g at 20 MPa/40 °C/150 min [33]; for several unspecified *H. lupulus* samples it was 2–9 g/100 g at 30–35 MPa/250–300 min [11,12].

Comparing the 10, 12.5, 15 and 37 MPa results (Table 1), an up to ~3-fold increase in SFE-CO_2_ extract yield was obtained in ~4-fold shorter τ (80 versus 300 min) when optimized P of 37 MPa was applied. Similarly, the extract with high TEAC_ORAC_ (1481 mg TE/g E) was produced at 37 MPa, remarkably reducing the τ of supercritical CO_2_-soluble antioxidant constituents’ recovery from hop pellets and augmenting its content by up to 334% as compared to 10–15 MPa treatments (Table 1). 

### 3.3. Bitter Acid Profile of Hop Extracts Obtained under Different SFE-CO_2_ Conditions

As reported in Table 4, α-and β-acids (soft resins) constituted the major portion of the hop SFE-CO_2_ extracts, depending on process parameters from ~72 to 92%. Non-polar solvent-soluble uncharacterized soft resins, hop essential oil components and waxy fraction could comprise the remaining 8–28% of the extract [2]. The percentage distribution of the individual constituents within the identified soft resins was as follows: adhumulone and humulone (25–36% of the total extract amount), colupulone (22–30%), cohumulone (20–25%) and finally, the sum of adlupulone and lupulone (13–15%). Considering extraction yields, 67.0–228.4 mg of these bitter acids was recovered from 1 g of hop pellets. The recovery of soft resins from hops gradually increased by 71%, increasing the P from 10 to 37 MPa (Table 4).

α-Bitter acids (humulones) comprised 54–64% of the total soft resins and were found in significantly varying amounts from ~391.0 to 594.9 mg/g E, corresponding to the recovery range of 36.5–137.6 mg/g HP (Table 4). Comparing these data with the manufacturer-provided α-bitter acid content in *Ella* hops (13.4 g/100 g HP), recovery efficiency of SFE-CO_2_ at 10, 12.5 and 15 MPa was 27, 76 and 98% of the declared content of humulones, respectively. However, higher P, up to 37 MPa, enabled substantial shortening of the process, from 300 to 80 min. The percentage of β-bitter acid (lupulones) in the soft resin was lower, ~45% at 10 MPa, 36% at 12.5 and 15 MPa and 40% at 37 MPa. 

Del Valle et al. [33] reported similar ratio of α-/β-acids (1.2–1.7/1) in oleoresins from *Nugget*, *Osorno* and *Elizalde Lake* variety hops. Humulone-rich (41%) antimicrobial extract was obtained from *Marynka* hop pellets by SFE-CO_2_ at 30 MPa/50 °C. [36]. It may be observed that the variations of SFE-CO_2_ conditions had lower effect on the concentration of β-acids than α-acids; the highest content of the former (345.0 mg/g E at 37 MPa) was only larger than the lowest one by 14% (302.9 mg/g E at 12.5 MPa). However, the recovery of β-acids was highly dependent on the process pressure: thus, up to 3-fold more lupulones (90.8 vs 30.5 mg/g HP) were recovered at 37 MPa than at lower pressures, which are most commonly used by the industry. 

Numerous beneficial bioactivities both in vitro and in vivo were reported for bitter acids-rich preparations [3]. In general, high TEAC_ORAC_ of Ella hop extracts (Table 1) was consistent with the previous reports showing prevalent links between strong in vitro oxygen radical scavenging capacity and high soft resin, mainly humulones, content in various hop extracts [37,38,39]. This is additionally supported by the calculated Pearson correlation coefficients (Supplementary Materials, Table S3), which indicate the significant positive correlation between the TEAC_ORAC_ and cumulative α and β-bitter acid amount, including the individual constituents within this group of bioactives (> 0.97 and *p* < 0.05 for values expressed in mg/g HP). 

### 3.4. Pigment Profile of Hop Extracts Obtained under Different SFE-CO_2_ Conditions

The quantitative composition of the selected supercritical-CO_2_ soluble pigments in hop extracts, namely chlorophylls and carotenoids, is reported in Table 4. Generally, the total amount of these pigments was very low compared to the bitter acid content and did not exceed 0.04% of the total extract mass. For example, the extract isolated at 10 MPa had only 20.7 μg/g of carotenoids, while chlorophylls were not detected (the color of this extract was pale yellow). The concentration of pigments in the extracts significantly increased by increasing P and at 37 MPa reached 166.2 and 235.1 μg/g E for chlorophylls and carotenoids, respectively. Consequently, the recovery of carotenoids at 37 MPa was 32, 4.2 and 2.3 times higher than at 10, 12.5 and 15 MPa, respectively. Although humulones are undoubtedly the major contributors to the overall TEAC_ORAC_ of the extracts, chlorophylls and carotenoids, both of which have a well-documented antioxidant potential in vitro and in biological systems [40], may also influence antioxidant capacity, which was higher for the extracts obtained at 15 and 37 MPa (Table 1). 

Chlorophyll A amounted to 45%, 67% and 88% of the sum of all chlorophylls at 12.5, 15 and 37 MPa, respectively (Table 4). The concentration of this compound significantly (~14-fold) increased from 10.6 μg/g E at 12.5 MPa to 146.1 μg/g E at 37 MPa, thus explaining the shift of SFE-CO_2_ extract color from yellow-orange to greenish-brown at elevated P. The chlorophyll B content variations were less pronounced, ranging from 12.8 to 20.5 μg/g across different SFE-CO_2_ extracts tested. Higher content of chlorophyll A compared to chlorophyll B (average ratio 7/3) was also characteristic for hydroethanolic extracts recently obtained from *Magnum*, *Marynka* and *Lubelski* hop varieties [41]. The presence of chlorophylls in SFE-CO_2_ extracts (without quantitative results) isolated from different hop varieties with pure CO_2_ [33,34] and with cosolvent ethanol [42] was previously reported in several articles. To the best of our knowledge, the effects of SFE-CO_2_ parameters on the quantitative composition of chlorophylls in the extracts and their recovery rates have not been reported.

### 3.5. Volatile Compound Profile of Hop Extracts Obtained under Different SFE-CO_2_ Conditions

SPME-GC×GC-TOF-MS was employed to analyze the differences in the volatile compound composition of *Ella* hop lipophilic extracts obtained under different SFE-CO_2_ conditions (Table 5). Quantitative assessment was based on the peak area (AU×10^7^), which is dependent on the amount of eluting from the GC column compound and is relevant for comparison purposes (Supplementary Materials, Table S4). 

The extract isolated at 10 MPa generated the highest total peak area, while pressure increase resulted in the lower peak area by 32 to 36%; however, it was not significantly different at 12.5, 15 and 37 MPa (Supplementary Materials, Table S4). These findings may be explained by the dilution of volatile and GC-detectable fraction by the nonvolatile components, which were recovered at remarkably higher yields at the higher pressures (Table 3). Comparing experimental mass spectra with various spectroscopic databases and retention indices with available literature data [24,25,26,27,28,29,30], 45 compounds belonging to the different chemical classes were identified in the tested SFE-CO_2_ extracts: monoterpene hydrocarbons (8), oxygenated monoterpenes (1), sesquiterpene hydrocarbons (11), oxygenated sesquiterpenes (1), alcohols (2), aldehydes (1), ketones (2), fatty acids (4) and esters (15). Dietz et al. recently reported the importance of different fractions of hop essential oil constituents on the sensory flavor characteristics [43]. Consequently, the composition of volatiles may be important for developing various applications of hop extracts.

Sesquiterpenes represented the major fraction of volatiles, accounting for 33.8–38.7% of the total quantified by GC volatiles. Sesquiterpene hydrocarbons such as β-humulene (6.3–7.0%), α-humulene (7.2–9.9%) and α-selinene (5.5–14.9%) with intense woody, spicy and pepper-like notes were found at the highest percentages in the headspace of extracts absorbed by SPME. These compounds were followed by herbal β-selinene (3.9–4.6%) and δ-cadinene (1.5–4.7%) and fruity α-ylangene (3.1–4.0%). Other identified sesquiterpene hydrocarbons individually contributed to less than 2% of the total GC peak area (Table 5). Yan and coworkers recently reported a high share of humulene, selinene and cadinene in the overall sesquiterpene content (43%) for the *Ella* hop essential oil obtained by hydrodistillation [26]. Both the percentage content (Table 5) and peak areas (Supplementary Materials, Table S4) indicate that sesquiterpene profile was dependent on extraction parameters, particularly P. For example, α-selinene significantly reduced from 14.9% (or 182 × 10^7^ AU) at 10 MPa to 5.5% (or 49 × 10^7^ AU) at 37 MPa. The extracts obtained at 10 and 12.5 MPa generated higher peak areas and percentage content of α-humulene (90–121 × 10^7^ AU; on average, 9.8%) than at 15 and 37 MPa (67–72 × 10^7^ AU; on average, 7.6%). The share of β-humulene in the headspace remained relatively stable, amounting to ~6.6% of the total GC peak area across the different extracts tested with no significant differences in AU at P > 12.5 MPa. Only negligible amounts (<0.5%) of oxygenated sesquiterpene caryophyllene oxide were found in extracts obtained up to 15 MPa (Table 5), while humulene epoxide II, previously reported in *Ella* hop essential oil at 0.4% [26], was not detected in these experiments. 

The percentage of monoterpenes in the headspace increased from 10.5% (or 127 × 10^7^ AU) to 17.6% (or 154 × 10^7^ AU) when P was raised from 10 to 37 MPa (Table 5; Supplementary Materials, Table S4). These changes were obtained due to the ~2-fold higher peak areas of herbal β-pinene (63 × 10^7^ AU; 7.0%) and spicy β-myrcene (56 × 10^7^ AU; 6.2%) at 37 MPa as compared to 10–15 MPa, both being the major identified monoterpene hydrocarbons. Linalool with the distinctively floral, citrus, woody and green notes was the only identified oxygenated monoterpene in *Ella* hop SFE-CO_2_ extracts. Its content did not significantly change at P > 12.5 MPa, ~2.0% of the total GC peak area equivalent to ~19 × 10^7^ AU across different extracts tested. As reported by Brendel et al., both myrcene and linalool are aroma-active constituents with the highest flavor dilution factor values among the other hop volatiles [29]. Recently, Duarte et al. suggested that the ratio of α-humulene/β-myrcene could be used as one of the parameters to differentiate between the aroma, bittering and dual-purpose hops [44]. For *Ella* hop SFE-CO_2_ extracts this ratio gradually decreased from 3.3/1 at 10 MPa to 2.5/1 at 12.5 MPa, 1.9/1 at 15 MPa and 1.3 at 37 MPa (Table 5; Supplementary Materials, Table S4), suggesting that P increase can shift the aroma profile of extracts from the dual-purpose hop typical characteristics towards the bitter hop-related ones [44]. The tunability of SFE-CO_2_ parameters to produce hop extracts with the desired organoleptic properties was previously demonstrated by Van Opstaele et al. as well [13,14]. 

Esters accounted for 12.2–22.3% of the identified headspace volatiles, with higher peak areas (Supplementary Materials, Table S4) and percentages (Table 4), thus more pronounced fruity, green and floral notes at P > 10 MPa. Recent aroma profile analysis of *Ella* hop essential oil also indicated the presence of various esters at a total of ~10% amount [26], which is comparable to the 10 MPa-derived SFE-CO_2_ sample. In agreement with the latter research [26], methyl-4-decenoate (5.5–11.1%) was the most major of the identified esters, followed by the pentyl 2-methylpropanoate (1.4–2.8%), methyl nonanoate (0.9–2.8%) and methyl octanoate (1.2–1.3%). Relatively high amounts (3–7%) of methyl-4-decenoate and pentyl 2-methylpropanoate were also characteristic of the essences obtained using hydrodistillation or SFE-CO_2_ from *Galaxy*, *Topaz*, *Vic Secret*, *Super Pride*, *Hallertau Tradition*, *Saphir*, *Spalter Select* and *Tettnanger* hops [26,45]. The content of other volatiles in the headspace was rather low (Table 5; Supplementary Materials, Table S4): alcohols (up to 2.3%), ketones (up to 1.3%), fatty acids (up to 1.0%) and aldehydes (up to 0.3%). 2-Undecanol (1.5–1.9%) with fresh, waxy and cloth notes was the most abundant compound within this group of volatiles, previously identified in Portuguese hops’ essential oils [24]. Fruity and waxy ketones 2-undecanone and 2-tridecanone comprised ~0.6% across different samples tested and were the predominant ketone fraction compounds for other hop varieties as well [26,45]. 

Besides organoleptic features, several identified major volatiles, particularly mono- and sesquiterpenes, are also known for their specific medicinal properties [5]. For example, direct radical scavenger myrcene shows antinociceptive and antimutagenic properties and acts protectively towards the inflammation and oxidation-induced brain, heart and skin tissue damages, including UVB-induced photoaging. β-Pinene exhibits antidepressant, sedative, supraspinal antinociceptive and antiproliferative activities and exerts antiviral properties against herpes simplex virus. Anticancer, anti-allergic and corticoid drug dexamethasone-like anti-inflammatory activities have been reported for humulene and its derivatives, while sedative, antidepressant, anticonvulsant and neuroprotective actions were additionally ascribed to linalool [5].

## 4. Conclusions

CCD-RSM was employed to optimize the P, T and τ in order to produce single variety *Ella* hop SFE-CO_2_ extracts with high yield (RFI) and strong ORAC (RFII). Statistically significant and reproducible models were obtained for both RFs, while τ, P and P^2^ together contributed to the >70% of the observed changes in extract yield and TEAC_ORAC_ values. Under the optimal extraction conditions (37 MPa, 43 °C, 80 min), SFE-CO_2_ amounted to 26.3 g/100 g of the greenish-brown lipophilic fraction with 867.9 mg/g E total bitter acid content, 1.5/1 α- to β-bitter acid ratio and 1481 mg TE/g E ORAC. Optimized SFE-CO_2_ also offered up to ~3-fold higher extraction yield and antioxidant recovery and also substantially shortened exhaustive extraction of bitter acids from hop pellets in comparison to the classical commercial one-stage SFE-CO_2_ at 10–15 MPa and 40℃. The cumulative amount of lipophilic pigments (carotenoids and chlorophylls) was negligible as compared to the bitter acid content (< 0.04% of the total extract mass). The major identified volatile compounds were monoterpene hydrocarbons β-pinene (up to 7.0%) and β-myrcene (up to 6.2%), sesquiterpene hydrocarbons β-humulene (up to 7.0%), α-humulene (up to 9.9%) and α-selinene (up to 14.9%), also unsaturated ester methyl-4-decenoate (up to 11.1%), providing fruity, herbal, spicy and woody odor to the *Ella* hop SFE-CO_2_ extracts. The variations in the headspace aroma profile under the different experimental conditions outlined the tunability of supercritical CO_2_ to produce extracts with different organoleptic properties utilizing the same hop variety. To the best of our knowledge, the recovery of bitter acid and antioxidant-rich fractions from dual-purpose *Ella* hops via SFE-CO_2_ has been reported for the first time. Due to the high concentration of valuable bioactive constituents and particular aroma characteristics, such single variety hop SFE-CO_2_ extracts could find multipurpose applications not only in brewing, but also in pharmaceutical, nutraceutical and cosmetics industries.

## Figures and Tables

**Table 1:** Yields and TEAC_ORAC_ of *Ella* hop SFE-CO_2_ extracts obtained under the different experimental conditions.

Samples	SFE-CO_2_ Parameters			
	SFE-CO_2_ I 10 MPa, 40 °C, 300 min	SFE-CO_2_ II 12.5 MPa, 40 °C, 300 min	SFE-CO_2_ III 15 MPa, 40 °C, 300 min	SFE-CO_2_ IV 37 MPa, 43 °C, 80 min
Extract yield, g/100 g HP	9.33 ± 0.46 ^a^	19.11 ± 0.88 ^b^	22.09 ± 0.76 ^c^	26.32 ± 0.46 ^d^
TEAC_ORAC_				
mg TE/g E	1251.79 ± 6.30 ^a^	1281.94 ± 41.22 ^a^	1515.16 ± 26.30 ^b^	1481.17 ± 50.87 ^b^
mg TE/g HP	116.79 ± 0.59 ^a^	244.98 ± 7.88 ^b^	334.70 ± 5.81 ^c^	389.84 ± 13.39 ^d^

E: extract; HP: hop pellets; SFE-CO_2_: supercritical carbon dioxide extraction; TEAC: Trolox equivalent antioxidant capacity; TE: Trolox equivalents; ORAC: oxygen radical scavenging capacity. Different superscript letters in the same row indicate significant differences (one-way ANOVA and Tukey‘s test *p* < 0.05).

**Table 2:** Central composite design matrix (levels of independent variables and variation levels) for SFE-CO_2_ optimization for extraction of non-polar constituents from *Ella* hops and values of observed responses.

Levels and Runs	SFE-CO_2_ Parameters			CO_2_ Density *, kg/m^3^	RFI: Extract Yield, g/100 g HP		RFII: TEAC_ORAC,_ mg TE/g HP	
	P, MPa	T, °C	τ, min		Experimental	Predicted	Experimental	Predicted
Center	35	50	60	899	23.24 ± 0.56	23.91	334.22 ± 14.15	344.65
Axial	25	50	60	834	20.15 ± 0.31	19.84	297.98 ± 19.92	295.34
Axial	35	40	60	935	25.12 ± 0.89	24.89	370.86 ± 12.90	360.63
Factorial	25	40	90	880	23.84 ± 1.32	23.60	351.05 ± 5.92	350.56
Factorial	25	60	30	787	13.85 ± 0.18	14.10	255.93 ± 7.42	253.41
Axial	35	50	90	899	25.74 ± 1.16	26.53	374.68 ± 5.43	368.99
Center	35	50	60	899	23.54 ± 0.56	23.91	338.53 ± 14.33	344.65
Center	35	50	60	899	24.22 ± 0.50	23.91	348.31 ± 14.74	344.65
Factorial	45	60	30	913	19.04 ± 0.62	19.38	304.38 ± 14.09	307.83
Factorial	45	40	90	975	27.57 ± 0.29	27.41	353.07 ± 19.91	358.56
Factorial	45	40	30	975	21.94 ± 0.47	22.15	304.98 ± 5.86	306.25
Axial	35	60	60	863	23.21 ± 0.54	23.01	355.29 ± 31.40	353.67
Factorial	25	60	90	787	22.72 ± 0.30	22.60	333.37 ± 9.10	335.06
Factorial	45	60	90	913	25.74 ± 0.66	25.44	347.98 ± 3.67	346.98
Center	35	50	60	899	23.14 ± 0.56	23.92	332.78 ± 14.09	344.65
Center	35	50	60	899	24.00 ± 0.55	23.91	345.15 ± 14.61	344.65
Axial	45	50	60	944	24.51 ± 0.23	24.39	335.77 ± 18.35	326.56
Center	35	50	60	899	24.52 ± 0.49	23.91	345.22 ± 2.21	344.65
Factorial	25	40	30	880	15.50 ± 0.27	15.90	251.79 ± 17.80	255.75
Axial	35	50	30	899	20.88 ± 1.28	19.65	308.17 ± 9.36	302.01

*: calculated using online Peace Software (http://www.peacesoftware.de/einigewerte/co2_e.html; accessed 15 March 2021). HP: hop pellets; SFE-CO_2_: supercritical carbon dioxide extraction; ORAC: oxygen radical scavenging capacity; P: extraction pressure; RF: response factor; *τ:* extraction time; T: extraction temperature; TEAC: Trolox equivalent antioxidant capacity. Yields are expressed per 100 g of unextracted HP; TEAC_ORAC_ values are expressed per g of unextracted HP.

**Table 3:** Analysis of variance of the regression parameters for the response surface quadratic models of *Ella* hop SFE-CO_2_ extract yield (RFI) and TEAC_ORAC_ (RFII).

Source	SS	df	MS	F-Value	*p*-Value
**RFI: Extract yield, g/100 g HP**					
Model	210.66	9	23.41	51.17	<0.0001 *
P (pressure, MPa)	51.71	1	51.71	113.06	<0.0001 *
T (temperature, °C)	8.85	1	8.85	19.36	0.0013 *
τ (time, min)	118.34	1	118.34	258.72	<0.0001 *
PT	0.4802	1	0.4802	1.05	0.3297 **
Pτ	2.98	1	2.98	6.51	0.0288 *
Tτ	0.3200	1	0.3200	0.6996	0.4224 **
P²	8.93	1	8.93	19.52	0.0013 *
T²	0.0030	1	0.0030	0.0066	0.9368 **
τ²	1.86	1	1.86	4.06	0.0716 **
Residual	4.57	10	0.4574		
Lack of Fit	3.03	5	0.6051	1.95	0.2399 **
Pure Error	1.55	5	0.3097		
Cor Total	215.23	19			
**RFII TEAC_ORAC,_ mg TE/g HP**					
Model	20,284.94	9	2253.88	35.20	<0.0001 *
P (pressure, MPa)	2435.47	1	2435.47	38.03	0.0001 *
T (temperature, °C)	121.10	1	121.10	1.89	0.1991 **
τ (time, min)	11,215.80	1	11,215.80	175.15	<0.0001 *
PT	7.70	1	7.70	0.1203	0.7359 **
Pτ	903.34	1	903.34	14.11	0.0037 *
Tτ	86.53	1	86.53	1.35	0.2721 **
P²	3123.23	1	3123.23	48.77	<0.0001 *
T²	429.66	1	429.66	6.71	0.0269 *
τ²	230.26	1	230.26	3.60	0.0872 **
Residual	640.35	10	64.03		
Lack of Fit	432.78	5	86.56	2.08	0.2196 **
Pure Error	207.57	5	41.51		
Cor Total	20,925.29	19			

*: significant; **: not significant; HP: hop pellets; SS: sum of square; df: degree of freedom; MS: mean square; F: Fisher value. SFE-CO_2_: supercritical carbon dioxide extraction; ORAC: oxygen radical scavenging capacity; P: extraction pressure; RF: response factor; *τ:* extraction time; T: extraction temperature; TEAC: Trolox equivalent antioxidant capacity.

**Table 4:** Bitter acid, chlorophyll and carotenoid content of *Ella* hop SFE-CO_2_ extracts obtained under the different experimental conditions.

Samples	SFE-CO_2_ Parameters			
	SFE-CO_2_ I 10 MPa, 40 °C, 300 min	SFE-CO_2_ II 12.5 MPa, 40 °C, 300 min	SFE-CO_2_ III 15 MPa, 40 °C, 300 min	SFE-CO_2_ IV 37 MPa, 43 °C, 80 min
**Bitter Acid Content**				
**α-Bitter acids**				
Cohumulone				
mg/g E	144.59 ± 2.89 ^a^	182.60 ± 8.47 ^b^	234.54 ± 0.24 ^c^	186.95 ± 8.03 ^b^
mg/g HP	13.49 ± 0.27 ^a^	34.90 ± 1.62 ^b^	51.81 ± 0.05 ^c^	49.21 ± 2.11 ^c^
Adhumulone + humulone				
mg/g E	246.37 ± 4.29 ^a^	349.19 ± 17.43 ^b^	360.15 ± 6.17 ^b^	335.92 ± 2.27 ^b^
mg/g HP	22.99 ± 0.40 ^a^	66.73 ± 3.33 ^b^	79.56 ± 1.36 ^c^	88.41 ± 0.60 ^d^
Total α-bitter acids				
mg/g E	390.96 ± 7.18 ^a^	531.79 ± 25.90 ^b^	594.69 ± 6.41 ^c^	522.87 ± 10.30 ^b^
mg/g HP	36.48 ± 0.67 ^a^	101.63 ± 4.95 ^b^	131.37 ± 1.42 ^c^	137.62 ± 2.71 ^c^
**β-Bitter acids**				
Colupulone				
mg/g E	217.38 ± 6.33 ^b^	186.47 ± 7.83 ^a^	212.82 ± 6.08 ^b^	225.94 ± 13.05 ^b^
mg/g HP	20.28 ± 0.59 ^a^	35.63 ± 1.50 ^b^	47.01 ± 1.34 ^c^	59.47 ± 3.43 ^d^
Adlupulone + lupulone				
mg/g E	109.69 ± 3.06 ^a^	116.45 ± 6.38 ^ab^	125.11 ± 1.68 ^b^	119.09 ± 6.39 ^ab^
mg/g HP	10.23 ± 0.29 ^a^	22.25 ± 1.22 ^b^	27.64 ± 0.37 ^c^	31.34 ± 1.68 ^d^
Total β-bitter acids				
mg/g E	327.07 ± 9.39 ^ab^	302.92 ± 14.20 ^a^	337.93 ± 4.40 ^b^	345.03 ± 6.66 ^b^
mg/g HP	30.51 ± 0.88 ^a^	57.88 ± 2.71 ^b^	74.65 ± 0.97 ^c^	90.81 ± 1.75 ^d^
**Total hop bitter acids**				
mg/g E	718.03 ± 16.57 ^a^	834.71 ± 40.10 ^b^	932.62 ± 2.01 ^c^	867.90 ± 16.96 ^b^
mg/g HP	66.99 ± 1.55 ^a^	159.51 ± 7.66 ^b^	206.02 ± 0.44 ^c^	228.43 ± 4.46 ^d^
**α-acid/β-acid ratio**	1.19	1.76	1.76	1.52
**Pigment Content**				
**Chlorophylls**				
Chlorophyll A				
μg/g E	-^ND^	10.60 ± 0.12 ^a^	41.19 ± 0.14 ^b^	146.13 ± 1.45 ^c^
μg/g HP	-^ND^	2.02 ± 0.02 ^a^	9.10 ± 0.03 ^b^	38.46 ± 0.38 ^c^
Chlorophyll B				
μg/g E	-^ND^	12.84 ±1.16 ^a^	20.48 ± 1.42 ^b^	20.10 ± 0.83 ^b^
μg/g HP	-^ND^	2.45 ± 0.22 ^a^	4.52 ± 0.31 ^b^	5.29 ± 0.22 ^c^
Total chlorophylls				
μg/g E	-^ND^	23.43 ± 1.04 ^a^	61.67 ± 1.28 ^b^	166.23 ± 2.28 ^c^
μg/g HP	-^ND^	4.48 ± 0.20 ^a^	13.62 ± 0.28 ^b^	43.75 ± 0.60 ^c^
**Carotenoids**				
Total carotenoids				
μg/g E	20.72 ± 1.18 ^a^	76.80 ± 3.39 ^b^	124.26 ± 0.59 ^c^	235.12 ± 1.33 ^d^
μg/g HP	1.93 ± 0.11 ^a^	14.68 ± 0.65 ^b^	27.45 ± 0.13 ^c^	61.88 ± 0.35 ^d^

-^ND^: not detected; E: extract; HP: hop pellets; SFE-CO_2_: supercritical carbon dioxide extraction; TEAC: Trolox equivalent antioxidant capacity; TE: Trolox equivalents; ORAC: oxygen radical scavenging capacity. Different superscript letters in the same row indicate significant differences (one-way ANOVA and Tukey’s test *p* < 0.05).

**Table 5:** Volatile compound composition (% of the total GC peak area) of *Ella* hop SFE-CO_2_ extracts obtained under different experimental conditions.

Compound	Exact Mass	RI_exp_	RI_lit_^A^	Odor Type: Description ^B,C^	SFE-CO_2_ Conditions			
					SFE-CO_2_ I 10 MPa, 40 °C, 300 min	SFE-CO_2_ II 12.5 MPa, 40 °C, 300 min	SFE-CO_2_ III 15 MPa, 40 °C, 300 min	SFE-CO_2_ IV 37 MPa, 43 °C, 80 min
**Monoterpenes, % of the total GC peak area**								
α-Pinene	136.1252	950	946 [24]	Herbal: herbal, fresh, terpenic, fruity, sweet, green, pine, earthy, woody	0.09 ± 0.00 ^a^	0.05 ± 0.00 ^a^	0.08 ± 0.02 ^a^	0.39 ± 0.03 ^b^
Camphene	136.1252	971	972 [25]	Woody: camphoreous, cooling minty, citrus, green, spicy	0.02 ± 0.01 ^a^	0.04 ± 0.00 ^a^	0.06 ± 0.03 ^a^	0.13 ± 0.05 ^a^
β-Pinene	136.1252	1000	989 [26]	Herbal: cooling, dry, woody, piney, spicy, eucalyptus	3.21 ± 0.07 ^a^	4.21 ± 0.05 ^b^	3.74 ± 0.21 ^b^	7.02 ± 0.08 ^c^
β-Myrcene	136.1252	1000	995 [26]	Spicy: peppery, terpenic, balsamic, metallic, musty, fruity, ethereal, herbaceous, woody	3.02 ± 0.01 ^a^	3.82 ± 0.04 ^b^	3.81 ± 0.04 ^b^	6.23 ± 0.17 ^c^
*p*-Cymene	136.1252	1015	1015 [27]	Terpenic: woody, fresh, terpenic, citrus, lemon, spicy	0.41 ± 0.01 ^c^	0.20 ± 0.00 ^b^	0.14 ± 0.00 ^a^	0.39 ± 0.00 ^c^
(*E*)-β-Ocimene	136.1252	1059	1052 [26]	Floral: herbal, mild, citrus, sweet, orange, lemon, tropical, green, woody	1.53 ± 0.02 ^d^	1.34 ± 0.01 ^c^	0.61 ± 0.00 ^a^	1.18 ± 0.00 ^b^
γ-Terpinene	136.1252	1074	1068 [25]	Terpenic: citrus, terpenic, herbal, oily, tropical, fruity, sweet	0.36 ± 0.04 ^a^	0.36 ± 0.00 ^a^	0.32 ± 0.00 ^a^	-^ND^
Terpinolene	136.1252	1104	1105 [25]	Herbal: fresh, woody, sweet, piney, citrus, anise	0.08 ± 0.01 ^a^	0.09 ± 0.00 ^a^	0.11 ± 0.02 ^a^	0.11 ± 0.00 ^a^
β-Linalool	154.1358	1119	1109 [26]	Floral: citrus, orange, floral, sweet, rose, woody, green	1.78 ± 0.08 ^a^	1.97 ± 0.04 ^ab^	1.97 ± 0.02 ^ab^	2.15 ± 0.14 ^b^
Total monoterpenes					10.50	12.08	10.84	17.60
**Sesquiterpenes, % of the total GC peak area**								
α-Copaene	204.1878	1375	1374 [28]	Woody: woody, spicy, earthy	0.18 ± 0.01 ^a^	0.24 ± 0.00 ^c^	0.21 ± 0.00 ^b^	-^ND^
α-Ylangene	204.1878	1401	1390 [24]	Fruity	3.14 ± 0.01 ^a^	3.53 ± 0.04 ^b^	3.47 ± 0.01 ^b^	4.01 ± 0.00 ^c^
β-Caryophyllene	204.1878	1438	1428 [24]	Spicy: musty, green, woody, clove, dry	0.64 ± 0.00 ^b^	0.67 ± 0.00 ^c^	0.61 ± 0.00 ^a^	0.76 ± 0.00 ^d^
Aromadendrene	204.1878	1439	1439 [28]	Sweet, dry	1.09 ± 0.01 ^a^	1.00 ± 0.07 ^a^	1.00 ± 0.00 ^a^	1.08 ± 0.00 ^a^
β-Humulene	204.1878	1457	1457 [27]	-^NR^	6.30 ± 0.48 ^a^	6.67 ± 0.30 ^a^	6.98 ± 0.02 ^b^	6.31 ± 0.22 ^a^
α-Humulene	204.1878	1504	1505 [25]	Woody: woody, spicy, clove	9.88 ± 0.02 ^b^	9.71 ± 0.08 ^b^	7.23 ± 0.00 ^a^	7.96 ± 0.78 ^a^
β-Selinene	204.1878	1514	1524 [24]	Herbal	-^ND^	4.30 ± 0.00 ^b^	4.61 ± 0.00 ^c^	3.88 ± 0.00 ^a^
α-Selinene	204.1878	1534	1533 [24]	Pepper, orange	14.86 ± 0.00 ^c^	10.55 ± 0.00 ^b^	5.90 ± 0.00 ^a^	5.49 ± 0.01 ^a^
δ-Cadinene	204.1878	1554	1556 [24]	Herbal: thyme, herbal, woody, dry	1.51 ± 0.00 ^a^	1.58 ± 0.03^a^	2.41 ± 0.01 ^a^	4.65 ± 0.08 ^b^
Calamenene	202.1722	1564	1562 [24]	Herbal, spicy	0.27 ± 0.00 ^a^	0.31 ± 0.00 ^b^	0.77 ± 0.00 ^c^	0.84 ± 0.00 ^d^
α-Calacorene	200.1565	1583	1590 [24]	Woody: dry, woody	0.21 ± 0.01 ^c^	0.17 ± 0.00 ^b^	0.20 ± 0.00 ^c^	0.13 ± 0.00 ^a^
Caryophyllene oxide	220.1827	1635	1617 [26]	Woody: sweet, fresh, dry, woody, spicy, fruity, sawdust, herbal	0.21 ± 0.00	-^ND^	0.41 ± 0.00	-^ND^
Total sesquiterpenes					38.29	38.73	33.80	35.11
**Alcohols, % of the total GC peak area**								
3-Methyl-2-buten-1-ol	86.0732	799	785 [24]	Fruity: sweet, fruity, alcoholic, green	0.20 ± 0.00 ^a^	0.44 ± 0.00 ^b^	0.50 ± 0.00 ^c^	-^ND^
2-Undecanol	170.1671	1314	1302 [24]	Waxy: fresh, waxy, cloth, sarsaparilla	1.74 ± 0.08 ^b^	1.89 ± 0.13 ^b^	1.80 ± 0.07 ^b^	1.48 ± 0.13 ^a^
Total alcohols					1.94	2.33	2.3	1.48
**Aldehydes, % of the total GC peak area**								
3-Methyl-2-butenal	84.0575	814	794 [24]	Fruity: sweet, fruity, pungent, nutty, almond, cherry	0.24 ± 0.01 ^b^	0.24 ± 0.05 ^b^	0.27 ± 0.06 ^b^	0.04 ± 0.01 ^a^
Total aldehydes					0.24	0.24	0.27	0.04
**Ketones, % of the total GC peak area**								
2-Undecanone	170.1671	1271	1294 [28]	Fruity: waxy, fruity, creamy, fatty, pineapple, orris, floral	0.63 ± 0.00 ^a^	0.60 ± 0.02 ^a^	0.64 ± 0.00 ^a^	0.58 ± 0.00 ^a^
2-Tridecanone	198.1984	1514	1504 [26]	Waxy: fatty, waxy, dairy, milky, coconut, nutty, herbal, earthy	0.67±0.00 ^a^	0.64±0.00 ^a^	-^ND^	-^ND^
Total ketones					1.30	1.24	0.64	0.58
**Esters, % of the total GC peak area**								
2-methylpropyl 2-methylpropanoate	144.1150	921	918 [26]	Fruity: ethereal, fruity, tropical, fruity, pineapple	0.08 ± 0.03 ^a^	0.09 ± 0.04 ^a^	0.16 ± 0.00 ^ab^	0.27 ± 0.01 ^b^
3-methylbutyl propanoate	144.1150	979	977 [26]	Fruity: sweet, fruity, apple, apple, raspberry, banana	0.56 ± 0.06 ^a^	0.69 ± 0.07 ^a^	0.59 ± 0.00 ^a^	1.05 ± 0.10 ^b^
Methyl hexanoate	130.0994	936	927 [24]	Fruity: fruity, pineapple, thinner, acetone	0.09 ± 0.00	0.10 ± 0.01	0.16 ± 0.00	0.11 ± 0.02
Pentyl 2-methylpropanoate	158.1307	1022	1020 [26]	Fruity: fruity, apple, banana, apricot, buttery	1.37 ± 0.02 ^a^	1.52 ± 0.18 ^a^	1.68 ± 0.09 ^a^	2.76 ± 0.05 ^b^
Methyl heptanoate	144.1150	1037	1030 [26]	Fruity: sweet, fruity, waxy, floral, berry, apple	0.56 ± 0.00 ^a^	0.65 ± 0.05 ^ab^	0.60 ± 0.04 ^a^	0.77 ± 0.06 ^b^
Methyl 6-methylheptanoate	158.1307	1096	1092 [24]	-^NR^	0.72 ± 0.02 ^a^	1.02 ± 0.00 ^b^	1.14 ± 0.00 ^c^	0.96 ± 0.05 ^b^
2-Methylbutyl 3-methylbutanoate	172.1463	1111	1113 [24]	Fruity: herbal, earthy, apple, green	-^ND^	-^ND^	0.52 ± 0.06	0.63 ± 0.09
Methyl octanoate	158.1307	1135	1130 [26]	Waxy: waxy, green, sweet, orange, aldehydic, vegetable, herbal	1.16 ± 0.01^a^	1.33 ± 0.08 ^a^	1.28 ± 0.06 ^a^	1.20 ± 0.05 ^a^
Hexyl 2-methylpropanoate	172.1463	1158	1151 [26]	Green: sweet, green, fruity, apple, pear, grape, ripe, berry	0.15 ± 0.06 ^a^	0.28 ± 0.00 ^abc^	0.61 ± 0.04 ^c^	0.36 ± 0.01 ^b^
Heptyl propanoate	172.1463	1206	1207 [24]	Floral: rose, apricot	0.61 ± 0.03 ^ab^	0.62 ± 0.01 ^ab^	0.67 ± 0.00 ^b^	0.56 ± 0.00 ^a^
Methyl 8-nonenoate	170.1307	1222	1218 [26]	-^NR^	-^ND^	0.55 ± 0.03 ^a^	0.50 ± 0.00 ^a^	0.49 ± 0.00 ^a^
Methyl nonanoate	172.1463	1238	1229 [26]	Fruity: sweet, fruity, pear, waxy, tropical, winey	0.91 ± 0.01	2.18 ± 0.12 ^bc^	2.83 ± 0.04 ^c^	2.13 ± 0.00 ^b^
Heptyl 2-methylpropanoate	186.1620	1255	1249 [26]	Fruity: fruity, sweet, green, warm, floral, tropical, chamomile, tea, green	0.39 ± 0.00	0.49 ± 0.00	0.53 ± 0.07 ^a^	0.42 ± 0.02 ^a^
2-Methylbutyl hexanoate	186.1620	1263	1246 [24]	Fruity: fruity, ethereal	0.06 ± 0.00	0.12 ± 0.00	-^ND^	-^ND^
Methyl 4-decenoate	184.1463	1322	1316 [26]	Fruity: fruity, pear, mango, fishy, peach, green	5.51 ± 0.04 ^a^	10.89 ± 0.22 ^c^	11.08 ± 0.27 ^c^	8.38 ± 0.06 ^b^
Total esters					12.17	20.53	22.35	20.09
**Fatty acids, % of the total GC peak area**								
2-Methylpropanoic acid	88.05240	778	762 [29]	Acidic: sour, cheesy, dairy, buttery, rancid, phenolic, fatty, sweaty	0.17 ± 0.00 ^a^	0.17 ± 0.02 ^a^	0.20 ± 0.02 ^a^	0.26 ± 0.05 ^a^
3-Methylbutanoic acid	102.0681	850	865 [29]	Cheesy: dairy, acidic, sour, pungent, fruity, fatty, sweaty, rancid	0.11 ± 0.01 ^a^	0.13 ± 0.00 ^a^	0.14 ± 0.00 ^a^	0.11 ± 0.01 ^a^
Heptanoic acid	130.0994	1089	1072 [24]	Cheesy: rancid, sour, cheesy, waxy, sweaty, fermented, pineapple, fruity	0.24 ± 0.00 ^a^	0.41 ± 0.01 ^c^	0.31 ± 0.02 ^b^	0.28 ± 0.02 ^ab^
Octanoic acid	144.1150	1189	1191 [30]	Fatty: fatty, waxy, rancid, oily, vegetable, cheesy	0.20 ± 0.00 ^a^	0.25 ± 0.00 ^c^	0.22 ± 0.00 ^b^	0.28 ± 0.00 ^d^
Total fatty acids					0.72	0.96	0.87	0.93

^A^: Retention indexes (RI) reported for RTX-5 or equivalent column (± 20 units compared to the calculated RI_exp_); [24] Martins et. al. *J. Chemom*., 2020, 34, e3285; [25] Rali et al. *Molecules*, 2007, 12, 3, 389–394; [26] Yan et al. *Food Chem*., 2019, 25, 15–23; [27] Frizzo et al. *Flavour Fragr. J.*, 2001, 16, 286–288; [28] Adams, R.P. Identification of essential oil components by gas chromatography/mass spectrometry, ed. 4.1. 2017; [29] Brendel et.al. *J. Agric. Food Chem.*, 2019, 67, 12044–12053; [30] Alissandrakis et al. *J. Agric. Food Chem.*, 2007, 55, 8152–8157. ^B^: Odor descriptions obtained from Pherobase database (https://www.pherobase.com/; accessed 16 March 2021); ^C^: Odor descriptions obtained from The Goodscent Company database (http://www.thegoodscentscompany.com/; accessed 16 March 2021); -^ND^: not detected; -^NR^: not reported. Different superscript letters in the same row indicate significant differences (one-way ANOVA and Tukey’s test *p* < 0.05).

## Data Availability

Not applicable.

## Supplementary Materials

The following are available online at https://www.mdpi.com/article/10.3390/antiox10060918/s1, Table S1: Fit statistics parameters for the quadratic models of *Ella* hop SFE-CO_2_ extract yield (RFI) and TEAC_ORAC_ (RFII); Table S2: Confirmation parameters for the quadratic models of *Ella* hop SFE-CO_2_ extract yield (RFI) and TEAC_ORAC_ (RFII) at the optimal conditions of the process (37 MPa, 43 °C, 80 min); Table S3: Analysis of correlation between TEAC_ORAC_ and phytochemical composition of *Ella* hop SFE-CO_2_ extracts obtained under the different experimental conditions; Table S4: Volatile compound composition (GC peak area arbitrary units × 10^7^) of *Ella* hop SFE-CO_2_ extracts obtained under different experimental conditions; Figure S1: Response surface 3D and 2D plots showing the effects of independent variables pressure (P), temperature (T) and time (τ) on the *Ella* hop SFE-CO_2_ extract yield (g/100 g HP); Figure S2: Response surface 3D and 2D plots showing the effects of independent variables pressure (P), temperature (T) and time (τ) on the *Ella* hop SFE-CO_2_ extract TEAC_ORAC_ (mg TE/g HP).
