# Supplementary material for: Optimized Supercritical CO2 Extraction Enhances the Recovery of Valuable Lipophilic Antioxidants and Other Constituents from Dual-Purpose Hop (Humulus lupulus L.) Variety Ella
Source: Antioxidants (Basel). 2021 Jun 6;10(6):918. doi: 10.3390/antiox10060918 (PMC8228826; doi:10.3390/antiox10060918)
Supplement: Supplementary file 1 [file antioxidants-10-00918-s001.zip › Kitryte_SupplementaryMat_Proofreading.pdf]

Optimized supercritical CO_2 extraction enhances the recovery of valuable lipophilic antioxidants and other constituents from dual-purpose hop (*Humulus lupulus* L.) variety *Ella*

Nóra Emilia Nagybákay[1], Michail Syrpas[1], Vaiva Vilimaitė[1], Laura Tamkutė[1], Audrius Pukalskas[1], Petras Rimantas Venskutonis[1] and Vaida Kitrytė[1,*]

Department of Food Science and Technology, Kaunas University of Technology, Radvilėnų Rd. 19, Kaunas LT-50254, Lithuania; nora.nagybakay@ktu.lt; michail.syrpas@ktu.lt; vaiva.vilimaite@ktu.lt; laura.tamkute@ktu.lt; audrius.pukalskas@ktu.lt; rimas.venskutonis@ktu.lt; vaida.kitryte@ktu.lt

* Correspondence: vaida.kitryte@ktu.lt; Tel.: (+370 37 300 153)

Supplementary Materials, Tables

Table S1. Fit statistics parameters for the quadratic models of *Ella* hop SFE-CO_2 extract yield (RFI) and $TEAC_{ORAC}$ (RFII).

Fit statistics parameters	RFI: Extract yield, g/100 g HP	RFII: $TEAC_{ORAC}$, mg TE/g HP
Standard deviation	0.6763	8.00
Mean	22.62	329.48
C.V. %	2.99	2.43
R^2	0.9787	0.9694
Adjusted R^2	0.9596	0.9419
Predicted R^2	0.8859	0.8538
Adequate precision	27.8325	20.4273

HP: hop pellets; SFE-CO_2: supercritical carbon dioxide extraction; ORAC: oxygen radical scavenging capacity; RF: response factor; TEAC: Trolox equivalent antioxidant capacity.

Table S2. Confirmation parameters for the quadratic models of *Ella* hop SFE-CO_2 extract yield (RFI) and $TEAC_{ORAC}$ (RFII) at the optimal conditions of the process (37 MPa, 43 °C, 80 min)

Confirmation parameters	RFI: Extract yield, g/100 g HP	RFII: $TEAC_{ORAC}$, mg TE/g HP
Predicted mean	26.7641	373.2250
Predicted median	26.7641	373.2250
Standard deviation	0.6763	8.0022
Predicted SE	0.7558	8.9434
95% PI (low)	25.0800	353.2980
Experimental data mean	26.3246	389.8444
Experimental standard deviation	0.4641	13.3877
Experimental SE	0.3282	6.6938
95% PI (high)	28.4483	393.1530
Desirability function coefficient*	0.4870	

HP: hop pellets; Predicted SE: standard deviation associated with the prediction of an individual observation; Experimental SE: standard error of experimentally obtained values, calculated as: $SE = standard\ deviation/\sqrt{n}$, where n is the number of replicated experiments. SFE-CO_2: supercritical carbon dioxide extraction; ORAC: oxygen radical scavenging capacity; PI: prediction interval; RF: response factor; TEAC: Trolox equivalent antioxidant capacity. *Multi-response desirability function coefficient was obtained under the following restraints in the Design-Expert 12 software: P and τ set as "minimize", T set as "in range", RFI set as "maximize" with the lower limit of 26 g/100 g HP, RFII set as "maximize" with the lower limit of 360 mg TE/g HP.

Table S3. Analysis of correlation between $TEAC_{ORAC}$ and phytochemical composition of *Ella* hop SFE-CO_2 extracts obtained under the different experimental conditions.

Phytochemical composition	Pearson correlation coefficients	
	$TEAC_{ORAC}$, mg TE/g E	$TEAC_{ORAC}$, mg TE/g HP
Total bitter acid content, mg/g E or HP	0.8664	0.9934**
α-Acid content, mg/g E or HP:	0.7473	0.9799*
Cohumulone	0.8240	0.9671*
Adhumulone + humulone	0.6462	0.9774*
β-Acid content, mg/g E or HP:	0.7570	0.9976**
Colupulone	0.4866	0.9912**
Adlupulone + lupulone	0.8871	0.9941**
Total carotenoid content, μg/g E or HP	0.7943	0.9064
Total chlorophyll content, μg/g of E or HP:	0.7479	0.8398
Chlorophyll A	0.6968	0.7958
Chlorophyll B	0.8803	0.9981**

E: extract; HP: hop pellets; SFE-CO_2: supercritical carbon dioxide extraction; ORAC: oxygen radical scavenging capacity; TEAC: Trolox equivalent antioxidant capacity; *: correlation is significant at the $p< 0.05$ level (two-tailed) Pearson correlation coefficients were calculated using GraphPad Prism 7.04 software (2017).

Table S4. Volatile compound composition (GC peak area arbitrary units × 10^7) of *Ella* hop SFE-CO_2 extracts obtained under different experimental conditions.

Compound	Exact Mass	RI_{exp}	RI_{lit}[A]	Odour type: description[B,C]	SFE-CO_2 conditions			
					SFE-CO_2 I 10 MPa, 40 °C, 300 min	SFE-CO_2 II 12.5 MPa, 40 °C, 300 min	SFE-CO_2 III 15 MPa, 40 °C, 300 min	SFE-CO_2 IV 37 MPa, 43 °C, 80 min
**Monoterpenes GC peak area AU, × 10^7**								
α-Pinene	136.1252	950	946 [24]	Herbal: herbal, fresh, terpenic, fruity, sweet, green, pine, earthy, woody	1.1±0.0[a]	0.5±0.1[a]	0.7±0.2[a]	3.5±0.3[b]
Camphene	136.1252	971	972 [25]	Woody: camphoreous, cooling minty, citrus green spicy	0.2±0.1[a]	0.4±0.0[a]	0.6±0.3[a]	1.2±0.5[b]
β-Pinene	136.1252	1000	989 [26]	Herbal: cooling, dry, woody, piney, eucalyptus, spicy	39.4±0.9[a]	39.1±0.5[a]	34.6±1.9[a]	63.2±0.7[b]
β-Myrcene	136.1252	1000	995 [26]	Spicy: peppery, terpenic, balsamic, metallic, musty, fruity, ethereal, herbaceous, woody	37.1±0.1[a]	35.4±0.4[a]	35.3±0.4[a]	56.1±1.5[b]
p-Cymene	136.1252	1015	1015 [27]	Terpenic: woody, fresh, terpenic, citrus, lemon, spicy	5.0±0.1[d]	1.9±0.0[b]	1.3±0.0[a]	3.5±0.0[a]
(*E*)-β-Ocimene	136.1252	1059	1052 [26]	Floral: herbal, mild, citrus, sweet, orange, lemon, tropical, green, woody	18.8±0.3[d]	12.4±0.1[c]	5.7±0.0[a]	10.6±0.0[b]
γ-Terpinene	136.1252	1074	1068 [25]	Terpenic: citrus, terpenic, herbal, oily, tropical, fruity, sweet	4.4±0.5[b]	3.3±0.0[b]	3.0±0.0[a]	–[ND]
Terpinolene	136.1252	1104	1105 [25]	Herbal: fresh, woody, sweet, piney, citrus, anise	1.0±0.1[a]	0.8±0.0[a]	1.0±0.2[a]	1.0±0.0[a]
β-Linalool	154.1358	1119	1109 [26]	Floral: citrus, orange, floral, sweet, rose, woody, green	21.8±1.0[b]	18.3±0.4[a]	18.2±0.2[a]	19.4±1.3[ab]
Total monoterpenes					127.5	111.2	99.0	153.8

**Sesquiterpenes GC peak area AU, × 10⁷**								
α-Copaene	204.1878	1375	1374 [28]	Woody: woody, spicy, earthy	2.2±0.1 [a]	2.2±0.0 [a]	1.9±0.0 [a]	- [ND]
α-Ylangene	204.1878	1401	1390 [24]	Fruity	38.5±0.1 [c]	32.7±0.4 [a]	32.1±0.1 [a]	36.1±0.0 [b]
β-Caryophyllene	204.1878	1438	1428 [24]	Spicy: musty, green, woody, clove, dry	7.9±0.0 [d]	6.2±0.0 [b]	5.7±0.0 [a]	6.8±0.0 [c]
Aromadendrene	204.1878	1439	1439 [28]	Sweet, dry	13.4±0.1 [b]	9.3±0.7 [a]	9.3±0.0 [a]	9.7±0.0 [a]
β-Humulene	204.1878	1457	1457 [27]	- [NR]	77.3±5.9 [b]	61.9±2.8 [ab]	64.6±0.2 [ab]	56.8±2.0 [a]
α-Humulene	204.1878	1504	1505 [25]	Woody: woody, spicy, clove	121.2±0.3 [c]	90.1±0.7 [b]	66.9±0.0 [a]	71.7±7.0 [a]
β-Selinene	204.1878	1514	1524 [24]	Herbal	- [ND]	39.9±0.0 [b]	42.7±0.0 [c]	34.9±0.0 [a]
α-Selinene	204.1878	1534	1533 [24]	Pepper, orange	181.6±0.0 [d]	97.4±0.0 [c]	54.6±0.0 [b]	49.4±0.1 [a]
δ-Cadinene	204.1878	1554	1556 [24]	Herbal: thyme, herbal, woody, dry	18.5±0.0 [b]	14.7±0.3 [a]	22.3±0.1 [c]	41.9±0.7 [d]
Calamenene	202.1722	1564	1562 [24]	Herbal, spicy	3.3±0.0 [b]	2.9±0.0 [a]	7.1±0.0 [c]	7.6±0.0 [d]
α-Calacorene	200.1565	1583	1590 [24]	Woody: dry, woody	2.6±0.1 [c]	1.6±0.0 [b]	1.9±0.0 [b]	1.2±0.0 [a]
Caryophyllene oxide	220.1827	1635	1617 [26]	Woody: sweet, fresh, dry, woody, spicy, fruity, sawdust, herbal	2.6±0.0 [a]	- [ND]	3.8±0.0 [b]	- [ND]
Total sesquiterpenes					469.0	358.8	312.8	316.0
**Alcohols GC peak area AU, × 10⁷**								
3-Methyl-2-buten-1-ol	86.0732	799	785 [24]	Fruity: sweet, fruity, alcoholic, green	2.5±0.0 [a]	4.1±0.0 [b]	4.6±0.0 [c]	- [ND]
2-Undecanol	170.1671	1314	1302 [24]	Waxy: fresh, waxy, cloth, sarsaparilla	21.3±1.0 [c]	17.5±1.2 [b]	16.7±0.7 [b]	13.3±1.2 [a]
Total alcohols					23.8	21.6	21.3	13.3
**Aldehydes GC peak area AU, × 10⁷**								
3-Methyl-2-butenal	84.0575	814	794 [24]	Fruity: sweet, fruity, pungent, nutty, almond, cherry	2.9±0.1 [b]	2.2±0.5 [b]	2.5±0.6 [b]	0.4±0.1 [a]
Total aldehydes					2.9	2.2	2.5	0.4
**Ketones GC peak area AU, × 10⁷**								

2-Undecanone	170.1671	1271	1294 [28]	Fruity: waxy, fruity, creamy, fatty, pineapple, orris, floral	7.7±0.0^{c}	5.6±0.2^{ab}	5.9±0.0^{b}	5.2±0.0^{a}
2-Tridecanone	198.1984	1514	1504 [26]	Waxy: fatty, waxy, dairy, milky, coconut, nutty, herbal, earthy	8.2±0.0^{b}	5.9±0.0^{a}	–ND	–ND
Total ketones					15.9	11.5	5.9	5.2
**Esters GC peak area AU, $\times 10^{7}$**								
2-methylpropyl 2-methylpropanoate	144.1150	921	918 [26]	Fruity: ethereal, fruity, tropical fruit, pineapple	1.0±0.4^{a}	0.83±0.37^{a}	1.48±0.00^{ab}	2.43±0.09^{b}
3-methylbutyl propanoate	144.1150	979	977 [26]	Fruity: sweet, fruity, apple, apple, raspberry, banana	6.9±0.7^{ab}	6.40±0.65^{a}	5.46±0.00^{a}	9.45±0.9^{b}
Methyl hexanoate	130.0994	936	927 [24]	Fruity: fruity, pineapple, thinner, acetone	1.1±0.0^{abc}	0.93±0.09^{ab}	1.48±0.00^{c}	0.99±0.18^{b}
Pentyl 2-methylpropanoate	158.1307	1022	1020 [26]	Fruity: fruity, apple, banana, apricot, buttery	16.8±0.3^{a}	14.1±1.67^{a}	15.55±0.83^{a}	24.84±0.45^{b}
Methyl heptanoate	144.1150	1037	1030 [26]	Fruity: sweet, fruity, waxy, floral, berry, apple	6.87±0.00^{a}	6.03±0.46^{a}	5.55±0.37^{a}	6.93±0.54^{a}
Methyl 6-methylheptanoate	158.1307	1096	1092 [24]	–NR	8.83±0.25^{a}	9.46±0.00^{a}	10.55±0.00^{b}	8.64±0.45^{a}
2-Methylbutyl 3-methylbutanoate	172.1463	1111	1113 [24]	Fruity: herbal, earthy, apple, green	–ND	–ND	4.81±0.56^{a}	5.67±0.81^{a}
Methyl octanoate	158.1307	1135	1130 [26]	Waxy: waxy, green, sweet, orange, aldehydic, vegetable, herbal	14.23±0.12^{b}	12.34±0.74^{ab}	11.85±0.56^{a}	10.80±0.45^{a}
Hexyl 2-methylpropanoate	172.1463	1158	1151 [26]	Green: sweet, green, fruity, apple, pear, grape, ripe, berry	1.84±0.74^{a}	2.60±0.00^{a}	5.65±0.37^{b}	3.24±0.09^{a}
Heptyl propanoate	172.1463	1206	1207 [24]	Floral: rose, apricot	7.48±0.37^{c}	5.75±0.09^{ab}	6.20±0.00^{b}	5.04±0.00^{a}
Methyl 8-nonenoate	170.1307	1222	1218 [26]	–NR	–ND	5.1±0.28^{b}	4.63±0.00^{ab}	4.41±0.00^{a}
Methyl nonanoate	172.1463	1238	1229 [26]	Fruity: sweet, fruity, pear, waxy, tropical, winey	11.16±0.12^{a}	20.22±1.11^{b}	26.19±0.37^{c}	19.17±0.00^{b}
Heptyl 2-methylpropanoate	186.1620	1255	1249 [26]	Fruity: fruity, sweet, green, warm, floral, tropical,	4.78±0.00^{a}	4.55±0.00^{a}	4.9±0.65^{a}	3.78±0.18^{a}

				chamomile, tea, green				
2-Methylbutyl hexanoate	186.1620	1263	1246 [24]	Fruity: fruity, ethereal	0.74±0.00^{a}	1.11±0.00^{b}	-ND	-ND
Methyl 4-decenoate	184.1463	1322	1316 [26]	Fruity: fruity, pear, mango, fishy, peach, green	67.59±0.49^{a}	100.18±2.04^{c}	102.54±2.5^{c}	75.43±0.54^{b}
Total esters					149.29	189.59	206.84	180.84
**Fatty acids GC peak area AU, × 10^{7}**								
2-Methylpropanoic acid	88.05240	778	762 [29]	Acidic: sour, cheesy, dairy, buttery, rancid, phenolic, fatty, sweaty	2.09±0.00^{a}	1.58±0.19^{a}	1.85±0.19^{a}	2.34±0.45^{a}
3-Methylbutanoic acid	102.0681	850	865 [29]	Cheesy: dairy, acidic, sour, pungent, fruity, fatty, sweaty, rancid	1.35±0.12^{a}	1.21±0.00^{a}	1.30±0.00^{a}	0.99±0.09^{a}
Heptanoic acid	130.0994	1089	1072 [24]	Cheesy: rancid, sour, cheesy, waxy, sweaty, fermented, pineapple, fruity	2.94±0.00^{a}	3.80±0.09^{b}	2.87±0.19^{a}	2.52±0.18^{a}
Octanoic acid	144.1150	1189	1191 [30]	Fatty: fatty, waxy, rancid, oily, vegetable, cheesy	2.45±0.00^{d}	2.32±0.00^{b}	2.04±0.00^{a}	2.52±0.00^{c}
Total fatty acids					8.83	8.90	8.05	8.37
**Other GC peak area AU, × 10^{7}**								
-^{NI}	112.0524	979			5.77±0.12^{b}	3.99±0.56^{a}	3.89±0.09^{a}	5.67±0.54^{b}
-^{NI}	194.1307	1045			14.84±0.25^{b}	11.69±0.09^{a}	21.84±0.00^{c}	27.90±0.09^{d}
-^{NI}	130.0994	1052			18.40±0.00^{d}	11.97±0.00^{b}	10.74±1.57^{ab}	8.19±0.00^{a}
-^{NI}	125.9809	1097			10.67±0.25^{a}	9.00±0.65^{a}	9.53±0.28^{a}	-ND
-^{NI}	152.1201	1174			-ND	-ND	-ND	4.05±0.36
-^{NI}	130.1358	1182			3.31±0.00^{c}	1.30±0.00^{a}	1.48±0.00^{b}	4.23±0.00^{d}
-^{NI}	130.0994	1255			5.27±0.49^{b}	4.64±0.00^{ab}	3.89±0.00^{a}	3.78±0.18^{a}
-^{NI}	268.2402	1296			17.91±0.00^{c}	13.54±0.46^{b}	13.23±0.56^{b}	10.44±0.54^{a}
-^{NI}	164.0837	1305			6.38±0.25	-ND	-ND	-ND

-^{NI}	150.1045	1331	9.81±0.00	-ND	-ND	-ND
-^{NI}	140.1201	1331	77.77±0.00	-ND	-ND	-ND
-^{NI}	268.2402	1340	33.12±0.00^{b}	59.27±0.00^{d}	50.72±0.28^{c}	0.99±0.00^{a}
-^{NI}	214.1933	1366	17.54±0.74^{b}	11.69±1.76^{a}	10.74±0.56^{a}	8.19±0.18^{a}
-^{NI}	146.0732	1467	33.12±0.00	-ND	-ND	-ND
-^{NI}	196.2191	1504	37.05±0.00^{c}	-ND	26.75±0.00 ^{a}	27.09±0.00^{b}
-^{NI}	134.1096	1524	54.95±0.25^{c}	22.26±0.00^{b}	22.49±0.50^{b}	18.27±0.00^{a}
-^{NI}	262.2661	1688	42.32±0.00^{c}	33.3±0.00^{b}	22.03±0.19^{a}	22.23±0.72^{a}
**Total GC peak area AU, × 10^{7}**			**1226.67 ±21.92^{b}**	**927.56±7.08^{a}**	**925.47±6.20^{a}**	**900.15±5.63^{a}**

AU: arbitrary units; [A]: retention indexes (RI) reported for RTX-5 or equivalent column (± 20 units compared to the calculated RI_{exp}); [24] Martins et.al. *J. Chemom.*, 2020, 34, e3285; [25] Rali et al. *Molecules*, 2007, 12, 3, 389-394; [26] Yan et al. *Food Chem.*, 2019, 25, 15-23; [27] Frizzo et al. *Flavour Fragr. J.*, 2001, 16, 286-288; [28] Adams, R.P. Identification of essential oil components by gas chromatography/mass spectrometry, ed. 4.1. 2017; [29] Brendel et.al. *J. Agric. Food Chem.*, 2019, 67, 12044-12053; [30] Alissandrakis et al. *J. Agric. Food Chem.*, 2007, 55, 8152-8157. [B]: Odour descriptions obtained from Pherobase database (https://www.pherobase.com/; accessed 16 March 2021); [C]: Odour descriptions obtained from The Goodscent Company database (http://www.thegoodscentscompany.com/; accessed 16 March 2021); -^{NI}: not identified; -ND: not detected; -^{NR}: not reported. Different superscript letters in the same row indicate significant differences (one-way ANOVA and Tukey`s test $p < 0.05$).

Supplementary Materials, Figures

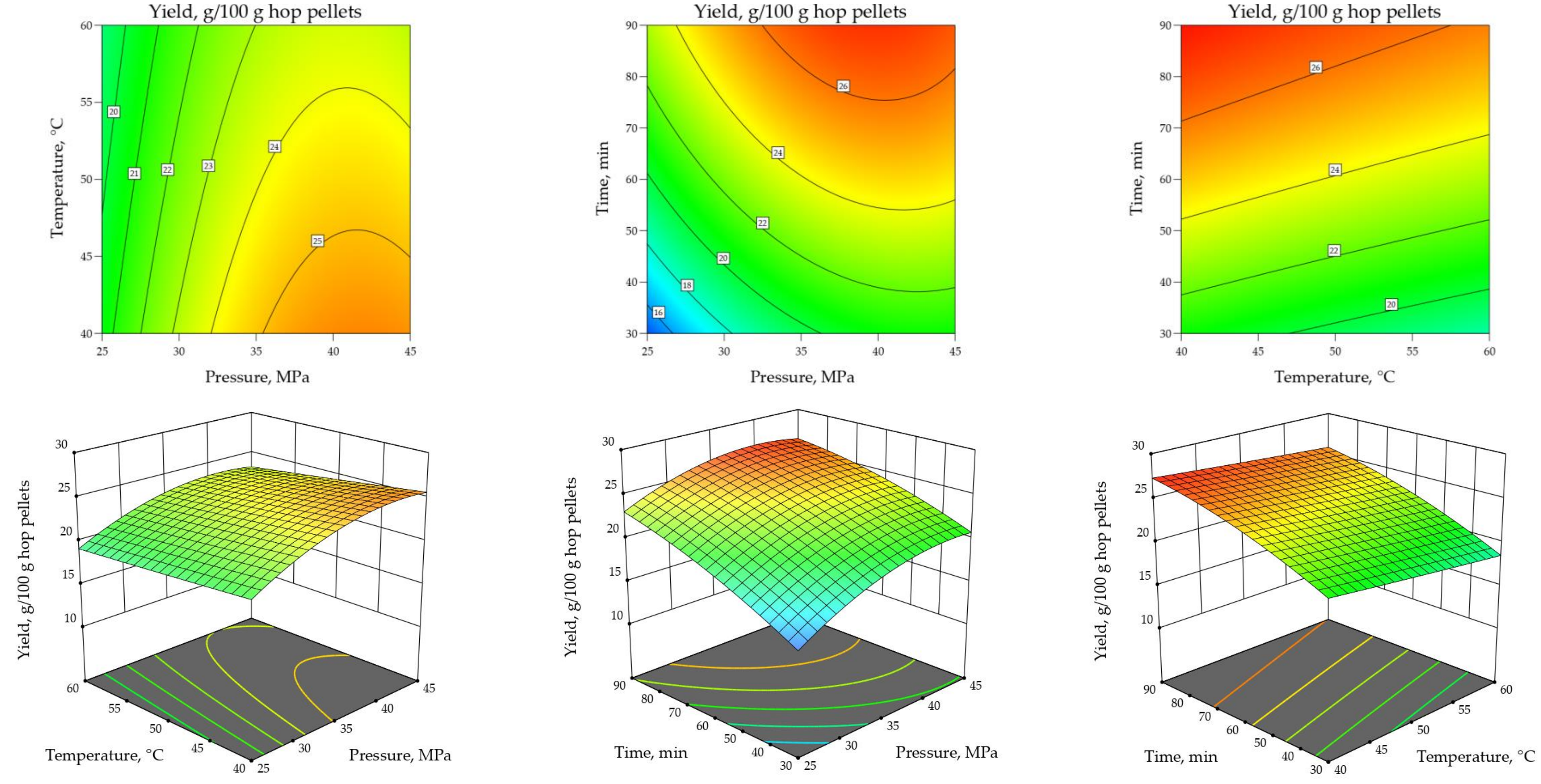

(a) effect of T and P (τ_{const}= 60 min) **(b) effect of τ and P (T_{const}= 50°C)** **(c) effect of τ and T (P_{const}= 35 MPa)**

Figure S1. Response surface 3D and 2D plots showing the effects of independent variables pressure (P), temperature (T) and time (τ) on the *Ella* hop SFE-CO_2 extract yield (g/100 g HP).

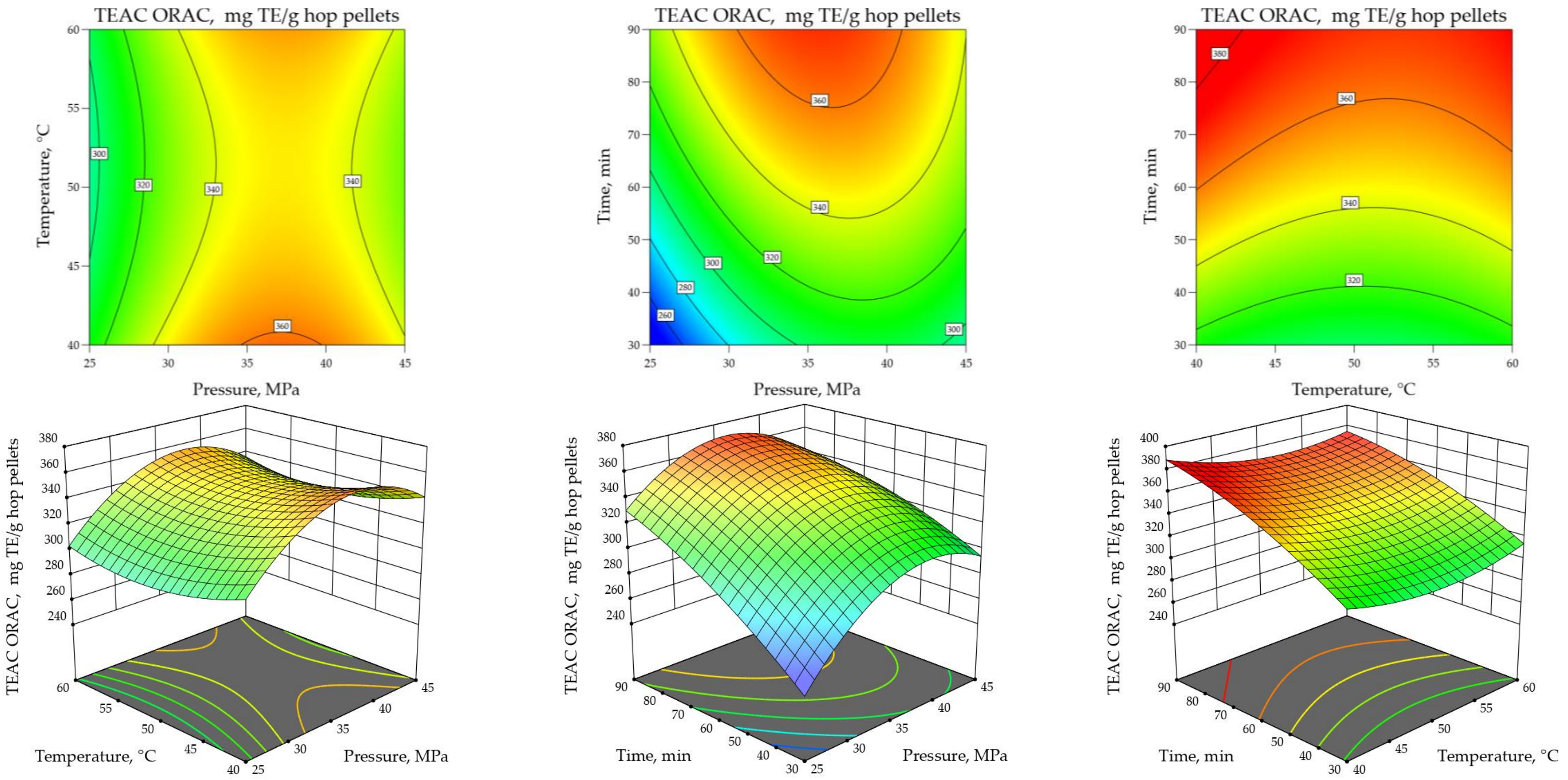

(a) effect of T and P (τ_{const}= 60 min) **(b) effect of τ and P (T_{const}= 50°C)** **(c) effect of τ and T (P_{const}= 35 MPa)**

Figure S2. Response surface 3D and 2D plots showing the effects of independent variables pressure (P), temperature (T) and time (τ) on the *Ella* hop SFE-CO_2 extract $TEAC_{ORAC}$ (mg TE/g HP).
